# Effect of traumatic brain injury on nicotine-induced modulation of dopamine release in the striatum and nucleus accumbens shell

**DOI:** 10.18632/oncotarget.24245

**Published:** 2018-01-13

**Authors:** Yuan-Hao Chen, Tung-Tai Kuo, Eagle Yi-Kung Huang, Yu-Ching Chou, Yung-Hsiao Chiang, Barry J. Hoffer, Jonathon Miller

**Affiliations:** ^1^Department of Neurological Surgery, Tri-Service General Hospital, National Defense Medical Center, Taipei, Taiwan, R.O.C; ^2^Graduate Institute of Computer and Communication Engineering, National Taipei University of Technology, Taipei, Taiwan, R.O.C; ^3^Department of Pharmacology, National Defense Medical Center, Taipei, Taiwan, R.O.C; ^4^School of Public Health, National Defense Medical Center, Taipei, Taiwan, R.O.C; ^5^Graduate Program on Neuroregeneration, Taipei Medical University, Taipei, Taiwan, R.O.C; ^6^Department of Neurosurgery, Case Western Reserve University School of Medicine, Cleveland, Ohio, USA

**Keywords:** traumatic brain injury, dopamine, striatum, nicotine desensitization, nucleus accumbens shell

## Abstract

**Background:**

Traumatic brain injury is associated with substantial alterations in reward processing, but underlying mechanisms are controversial.

**Objective:**

A better understanding of alterations in dopamine (DA) release patterns from the dorsal striatum and nucleus accumbens shell (NAc) may provide insights into posttraumatic reward pathology.

**Materials and Methods:**

The patterns of DA release with or without exposure to nicotine in brain slices with striatum and NAc, isolated from Sprague-Dawley rats with 6 psi fluid percussion (FPI) or sham injury were analysis by using fast-scan cyclic voltammetry. Tonic and phasic DA releases were assessed using single pulse and 10 pulses at 25 Hz, respectively. DA release relative to stimulation intensity, frequency, number of pulses, and paired-pulse facilitation was evaluated to determine release probability and response to bursting.

**Results:**

There was a profound suppression in tonic DA release after nicotine desensitization after FPI, and the input/output curve for the DA release based on stimulation intensity was shifted to the right. FPI was associated with a significant decrease in frequency-dependent DA release augmentation, DA release induced by high frequency stimulation trains, and DA release in response to paired-pulse facilitation. The effect of nicotine desensitization was similar in FPI and sham-injured animals, although significantly smaller after FPI. Nicotine desensitization–induced differences between phasic and tonic release concentrations that contrasted with the reward-related signals then became less prominent in NAc after FPI.

**Conclusions:**

TBI blunts DA release from mesolimbic reward centers, and more intense stimuli are required to produce context-dependent DA release sufficient to have a physiological effect.

**Implications:**

The nicotine desensitization-related suppression in tonic DA release was profound with right-ward shift of the input/output curve for DA release after FPI. FPI was associated with a significant decrease in frequency-dependent DA release augmentation, DA release induced by high frequency stimulation trains, and DA release in response to paired-pulse facilitation. Nicotine desensitization–induced differences between phasic and tonic release concentrations that contrasted with the reward-related signals then became less prominent in NAc after FPI. TBI thus blunts DA release from mesolimbic reward centers, and more intense stimuli are required to produce context-dependent DA release sufficient to have a physiological effect.

## INTRODUCTION

Traumatic brain injury is associated with alterations in physiological reward processing that may explain behavioral changes and substance abuse often seen in this population [1, 2]. 1. Head injury patients without mental illness or substance abuse-related service utilization prior to injury showed increased rates of substance use dependence (SUD) and depression compared with community controls [3, 4]. These patients have a 4.5 odds ratio of substance abuse within the first year post-injury, dropping to 1.4 at 25–36 months post-injury [5]. Moreover, soldiers with mild TBI were 2.6 times and those with a moderate TBI were 5.4 times more likely to be discharged for alcoholism or drug use [6, 7].

The striatal dopamine (DA) system is crucial for habit formation [8, 9], and the mesocorticolimbic system is strongly linked to reward behavior [10]. Thus, analysis of DA release from different systems after exposure to addictive agents has clarified the mechanism of the reward behavior process [11]. Investigation of DA release may provide an explanation for substance abuse formation among certain patient groups, such as patients with traumatic brain injury (TBI) [12] or stroke [13, 14].

Several mechanisms induced by TBI that affect neurotransmission especially dopamine (DA) transmission were elucidated, involving the systemic and microenvironmental effects of neuroinflammation induced after the initial insult in TBI [15, 16] and playing a crucial role in secondary neurodegeneration [15]. TBI induces time-dependent upregulation of apoptosis-related genes during the 3–48 h post-injury period that include inflammatory cytokines such as interleukin 1 (IL-1), and tumor necrosis factor (TNF) as well as prostaglandin (PG) synthases and cyclooxygenase (COX) 1 and 2. These may contribute to inflammation in the brain [17]. Thus, the neurodegenerative changes in expression of apoptosis-related genes after TBI may be associated with inflammatory responses [18]. Moreover, significant decreases of TH-positive expression in the surviving dopaminergic neurons of the SN pars compacta (SNpc) and increased a-synuclein accumulation in inflammation-infiltrated SN of rats exposed to chronic TBI were shown [16]. These phenomena may be a pathological link between chronic effects of TBI and PD symptoms and may be one of critical mechanisms of dopamine transmission impairment after TBI [15].

Previous studies have demonstrated that low-frequency stimulation evokes DA release suppression and that higher frequency enhancement is caused by nicotine desensitization and blockade through the α4β2 receptor in the striatum [19–21]; furthermore, frequency-dependent augmentation was found in the nucleus accumbens (NAc) shell [22, 23].

There are two different patterns of DA release *in vivo* that have distinct physiological effects: tonic low-frequency release (5–10 Hz), which represents baseline DA neuron firing, and phasic burst-like release (20–100 Hz), typically associated with reinforcers or reward predictors [24]. Paired-pulse stimulation to determine the interaction between firing frequency and dynamic release probabilities at varying interpulse intervals can provide insight into efficacy of reinforcement [25]. Paired-pulse facilitation in the presence of nicotine and a nicotine inhibitor (Mec) has been observed in both the striatum and NAc shell with intervals of less than 80 ms (>12.5 Hz), which suggests that nicotine acts as a switch of the frequency filter [24]. By enhancing the contrast in DA release when DA neuron activity switches from tonic to phasic firing in response to salient primary rewards or conditioned reward predictions [26], nicotine might enhance the reinforcement efficacy of any reward-related stimuli [24].

Application of different stimulation frequencies can be used to mimic tonic and phasic DA release from slices *in vitro* [27], and this can provide insight into the effect of fluid-percussion injury on these processes, especially the effect of nicotine on dynamic DA release probability and comparison of tonic and phasic reinforcing efficacy of the reward-stimuli response [28]. In this study, we used fast-scan cyclic voltammetry (FSCV) study to determine DA release patterns in brain slices with fluid percussion injury (FPI) to detect DA tonic release, frequency-dependent responses, the reaction to increments in high-frequency stimulation (HFS) trains, and paired-pulse ratios in different paired-pulse intervals in response to nicotine desensitization to determine the effect on impairment of reward behavior after head injury. By using paired pulses at different stimulation intervals (from 10 to 80 μsec), the paired-pulse release ratios (p2/p1) could be measured to determine the interaction between firing frequency and dynamic release probability.

## RESULTS

### Tonic DA release suppression related to nicotine desensitization and blockade was exacerbated after FPI (Figure 1)

**Figure 1:**
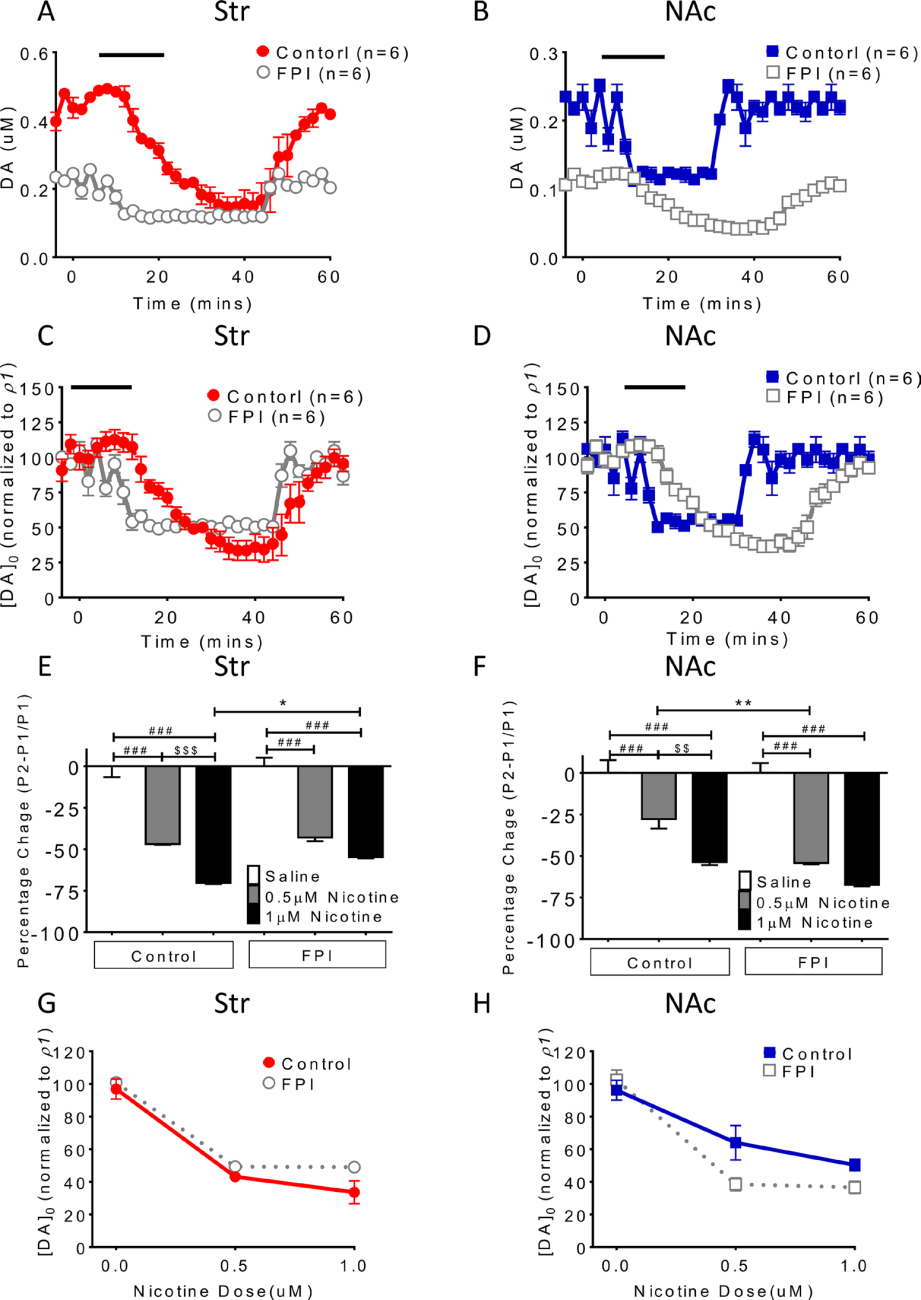
Nicotine desensitization and blockade–related DA release suppression is exacerbated after FPI (**A**) The nicotine desensitization–related suppression of tonic DA (single pulse/25 Hz evoked) was observed in brain slices (gray open circle). (**B**) DA tonic release was suppressed after FPI, and FPI further suppressed nicotine desensitization–related DA release in the shell portion. The suppression proportion was calculated using the normalized concentration of DA (Y axis [DA]_0_ (normalized to control P1): evoked signal in the striatum or shell/P1 in control striatum or shell; P1: mean control concentration evoked using 1 pulse). Similar DA release suppression was observed after nicotine blockade, and further suppression was noted after FPI in the (**C**) striatum and (**D**) NAc shell. The percentage expresses a change in a variable. It represents the change between a baseline value and a subsequent one. Value = (subsequent − baseline)/baseline × 100%. (**E**) The DA release concentration was lower in the FPI rats than in the control rats in the striatum. (**F**) In the NAc shell, suppression related to nicotine desensitization was more profound compared with that in the control group (particularly at 0.5 uM *p* < 0.01, one-way ANOVA followed by a Bonferroni post hoc test for multiple comparisons, Control + Nicotine vs. FPI + Nicotine: *P* < 0.05^*^, *P* < 0.01^**^; Saline vs. other groups: *p* < 0.001^###^ at Control and FPI groups; 0.5µM Nicotine vs. 1µM Nicotine: *P* < 0.01^$$^, *P* < 0.001^$$$^ at Control and FPI groups). The dose-dependent effect of nicotine desensitization on DA suppression became less apparent after FPI in the striatum (**G**), and similar results were observed in the NAc shell (**H**).

The baseline DA release concentration was lower in the FPI rats than in the control rats, and nicotine desensitization–related suppression of tonic DA release (single pulse/25 Hz evoked) was exacerbated in brain slices (Figure 1A and 1B). In the NAc shell, FPI suppressed tonic DA release to a greater degree relative to baseline and further attenuated suppression of DA release related to nicotine desensitization (Figure 1B and 1D). Furthermore, in both striatum and NAc shell slices, a similar suppression pattern of tonic DA release was observed after nicotine desensitization and blockade (Figure 1C and 1D). The suppression proportion (percentage) was calculated and plotted, revealing low suppression in the striatum (Figure 1E) and profound suppression in the NAc shell (Figure 1F). DA release was further suppressed by FPI. The dose-dependent effect of nicotine desensitization on DA release was diminished after FPI to the striatum (Figure 1G). A similar result was observed in the NAc shell (Figure 1H). Nicotine desensitization and blockade markedly suppressed tonic DA release in injured brain slices; however, the concentration difference between the baseline and desensitization conditions was small because of the low overall suppression of DA release after FPI in the striatum (Figure 1E and 1G) but more profound suppression in the NAc shell and Figure 1F and 1H).

### The I/O curve for the dopamine release pattern related to nicotine desensitization was shifted right after FPI (Figure 2)

**Figure 2:**
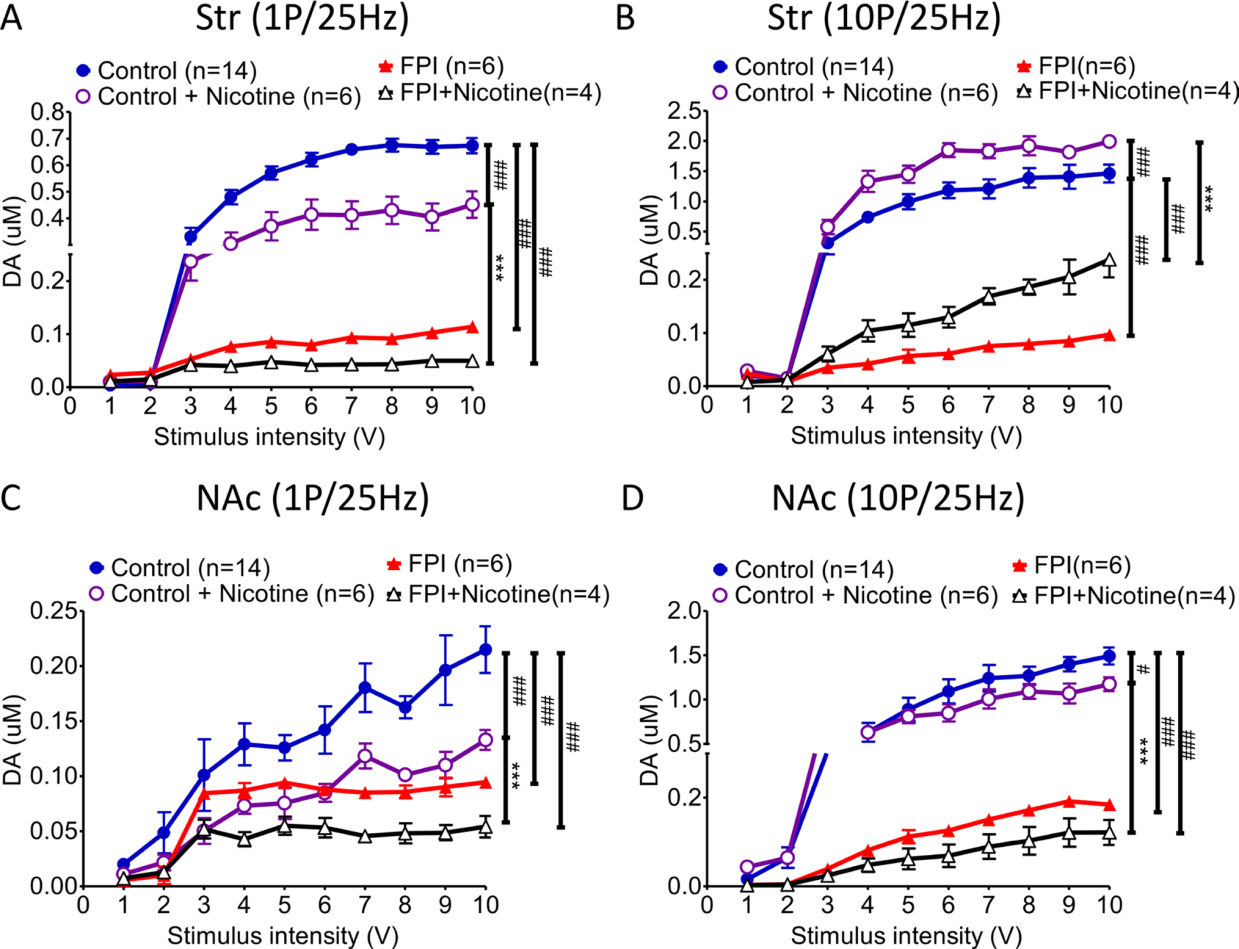
DA release evoked by different stimulation intensities (from 1 to 10 V) was plotted in an I/O curve (**A**) The tonic release of DA in the striatum decreased significantly after FPI, and nicotine desensitization–related tonic release suppression was still observed after FPI (FPI: red triangle vs. FPI + nicotine: open triangle). (**B**) Although the phasic release concentration was generally lower after FPI, the nicotine desensitization–induced phasic release enhancement in the striatum was still observed after FPI. (**C**) Tonic release in the NAc shell was low and flat after FPI, and the concentration was further suppressed by nicotine desensitization in the FPI brain slices (FPI + nicotine: open triangle). (**D**) Phasic release substantially increased under control and nicotine desensitization conditions, but after FPI, all of the change in phasic release was severely attenuated in the NAc shell portion. Nicotine desensitization further suppressed phasic release in the shell sections (black open triangle). Two-way ANOVA was followed by a Bonferroni test with 10V, Control + Nicotine vs. FPI + Nicotine, *P* < 0.001^***^; Control vs. other groups, *p* < 0.01^##^, *p* < 0.001^###^.

The I/O curve represents DA release evoked at various stimulation intensities (from 1 V to 10 V). Nicotine desensitization suppressed tonic release (Figure 2A; FPI: red triangle vs. FPI + nicotine: open triangle); but enhanced phasic release in striatal slices could still be found after FPI with the curve shifting to the right due to the evoked signal being diminished by FPI (Figure 2B). The difference between tonic and phasic DA release in the striatum was decreased after FPI. In contrast, either tonic release or phasic release was suppressed by nicotine desensitization in the NAc shell (Figure 2C and 2D), and the both tonic and phasic I/O curves were shifted to the right because of the weak signal after FPI. In summary, although the DA signals were weak after FPI, suppression of tonic release (Figure 2A) and enhancement of phasic release (Figure 2B) may have enhanced the DA response because salient stimuli that cause burst firing remained in the striatum. However, both tonic or phasic release in the NAc shell was significantly suppressed after FPI (Figure 2C and 2D).

### Frequency-dependent augmentation was affected by FPI (Figure 3)

**Figure 3:**
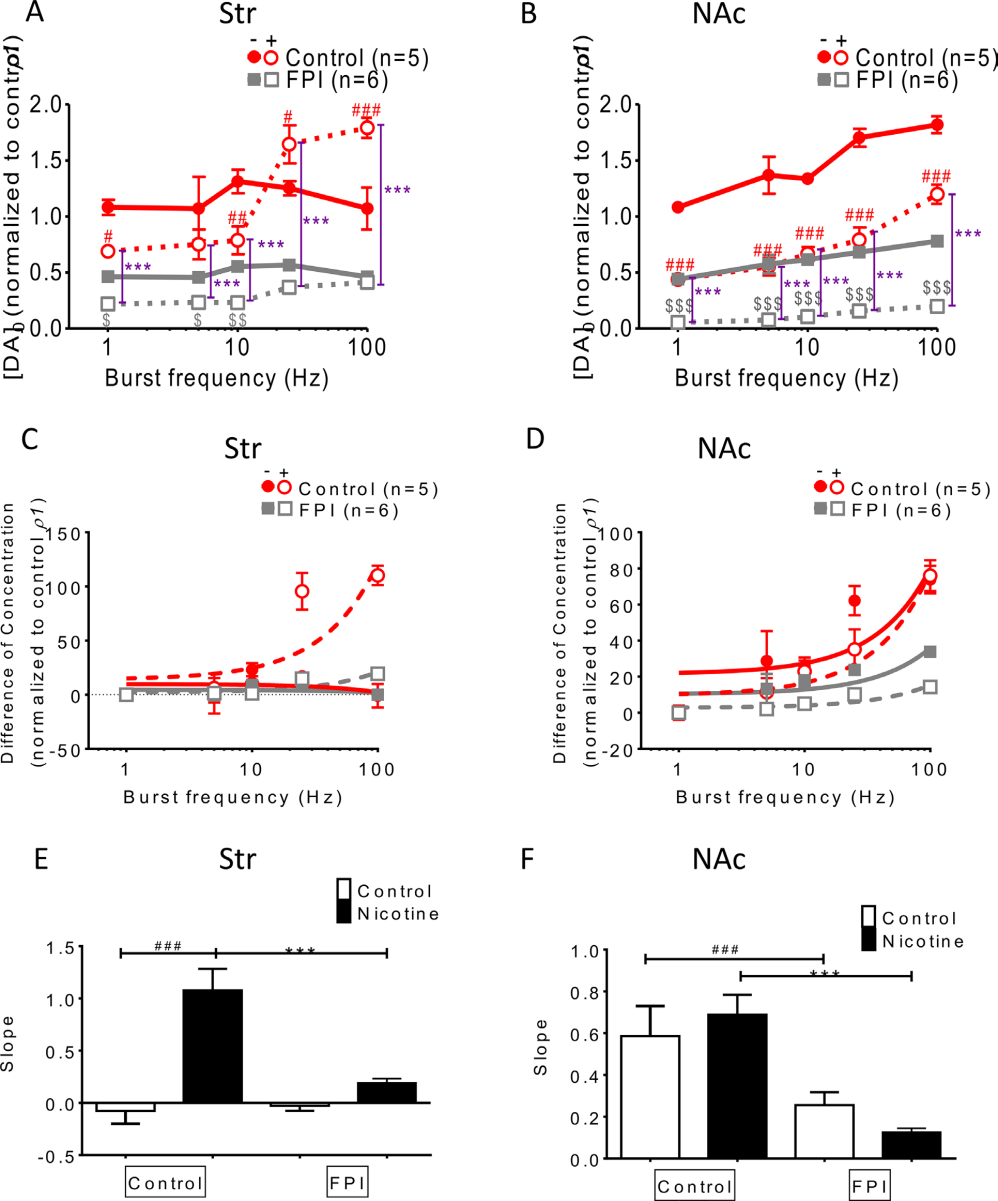
Frequency-dependent augmentation was affected by FPI (**A**) DA release was enhanced by bursting frequencies (more than 10 Hz) in striatal slices infused with nicotine, and this frequency-dependent augmentation was suppressed after FPI. (**B**) Frequency-dependent augmentation was found in both the control and nicotine infusion slices, and was suppressed after FPI. (**C**) The difference in concentrations between single-pulse (tonic) and bursting-stimulation (phasic) release is plotted versus stimulation frequency (in log units) in the striatum and NAc shell portion in (C) and (**D**), respectively. (**E**) The slopes of the equation of concentration difference, which shows the release probability of DA in each group, were plotted, revealing that the slope under nicotine infusion in the striatum decreased after FPI. (**F**) The slopes for the shell portion were compared, indicating that the release probabilities were reduced by FPI, and further suppression was observed in the FPI slices under nicotine infusion. (DA concentration normalization: one-way ANOVA followed by a Bonferroni post hoc test for multiple comparisons, Control + Nicotine vs. FPI + Nicotine, *P* < 0.001^***^; Control vs. Control + Nicotine, *p* < 0.05^#^, *p* < 0.01^##^, *p* < 0.001^###^; FPI vs. FPI + Nicotine, *p* < 0.05^$^, *p* < 0.01^$$^, *p* < 0.001^$$$^).

DA release was enhanced using bursting frequencies (more than 10 Hz) under nicotine infusion in the normal striatum, but this phenomenon was suppressed after FPI (Figure 3A). In the NAc shell, frequency-dependent augmentation facilitated DA release in both the control and nicotine infusion slices; this augmentation was diminished by FPI because it generally suppressed DA release (Figure 3B). The difference in concentration between single-pulse (tonic) and bursting-stimulation (phasic) release was plotted versus stimulation frequency (in log units) in the striatum and NAc shell portions (Figure 3C and 3D). The slope of the equation of concentration difference, used for calculating the DA release probability, was compared among groups (Figure 3E and 3F) [29]. The slope for striatal slices infused with nicotine decreased after FPI (Figure 3E), and a similar result was observed for NAc shell portion (Figure 3F). These data indicate that the enhancement of DA release through frequency-dependent augmentation in striatal slices infused with nicotine was diminished overall after FPI, and the enhancements became undistinguishable after FPI in the NAc shell portion.

### DA release increment induced by multiple HFS trains was suppressed overall by FPI (Figure 4)

**Figure 4:**
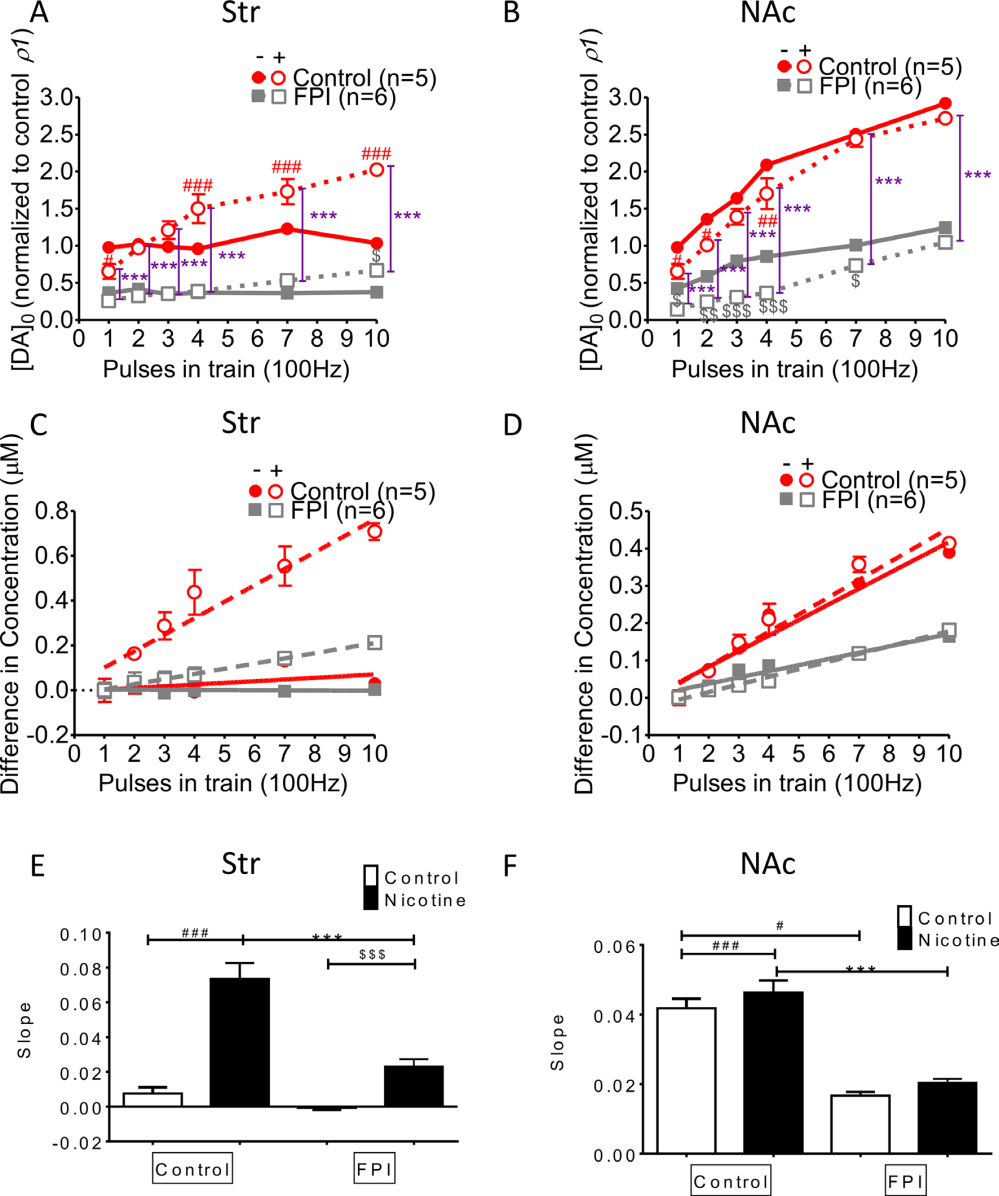
DA release enhanced by increases in HFS trains was affected by FPI (**A**) DA release was enhanced with an increasing difference in the HFS trains under nicotine desensitization. DA release was profoundly suppressed after FPI, but the enhancement in release along with increment in HFS trains was still induced by nicotine infusion. (**B**) An increase in DA release was observed under both control and nicotine desensitization conditions in the NAc shell portion; generalized suppression was observed after FPI (FPI only: gray solid square; FPI with nicotine infusion: gray open square). (**C**) Linear regression for the concentration difference between multiple trains and a single train of HFS in the striatum. Augmentation occurred only under nicotine infusion, and further suppression (shallowness of the slope) was observed only in the FPI slices under nicotine infusion. (**D**) Linear regression of the concentration difference in the shell portion revealed that the augmentation effect of HFS trains was prominent under nicotine infusion. However, this augmentation effect resulting from an increment in HFS trains was not distinguishable between the control and nicotine infusion groups after FPI. (**E**) The slope of the linear regression of the difference in concentrations evoked using different HFS trains in the striatum was plotted, and the nicotine desensitization–induced augmentation effect was attenuated after FPI. (**F**) The slope revealed no difference between the control and nicotine infusion after FPI. (DA concentration normalization: one-way ANOVA followed by a Bonferroni post hoc test for multiple comparisons, Control + Nicotine vs. FPI + Nicotine, *P* < 0.001^***^; Control vs. Control + Nicotine, *p* < 0.05^#^, *p* < 0.01^##^, *p* < 0.001^###^; FPI vs. FPI + Nicotine, *p* < 0.05^$^, *p* < 0.01^$$^, *p* < 0.001^$$$^).

DA release was enhanced with increments in HFS trains in striatal slices infused with nicotine, but this incremental DA release was diminished after FPI because it profoundly suppressed DA release (Figure 4A). Increasing DA release was observed in both the control and nicotine desensitization conditions in the NAc shell portion, and generalized suppression was observed after FPI (Figure 4B; FPI only: gray solid square; FPI with nicotine infusion: gray open square). We calculated the difference in concentrations between multiple trains of HFS (Figure 4C and 4D) and a single train of HFS by using linear regression to assess the slope for release probability (Figure 4E and 4F) [29]. Augmentation occurred only with nicotine infusion, and further suppression (indicated by the shallowness of the slope) was observed in the slices infused with nicotine after FPI (Figure 4C). In the shell portion, the augmentation effect of HFS trains was prominent under nicotine infusion. However, this augmentation effect resulted from incremental changes of HFS trains, and it was indistinguishable for control and nicotine-infused slices after FPI (Figure 4D). We plotted the slope of the linear regression for the difference in concentrations evoked by different HFS trains in the striatum (Figure 4E). Under the nicotine desensitization conditions, HFS trains induced an augmentation effect, which was attenuated after FPI in the striatum and became virtually absent in the NAc shell after FPI (Figure 4F).

### Nicotine desensitization–related paired-pulse facilitation at high frequencies was suppressed by FPI (Figure 5)

**Figure 5:**
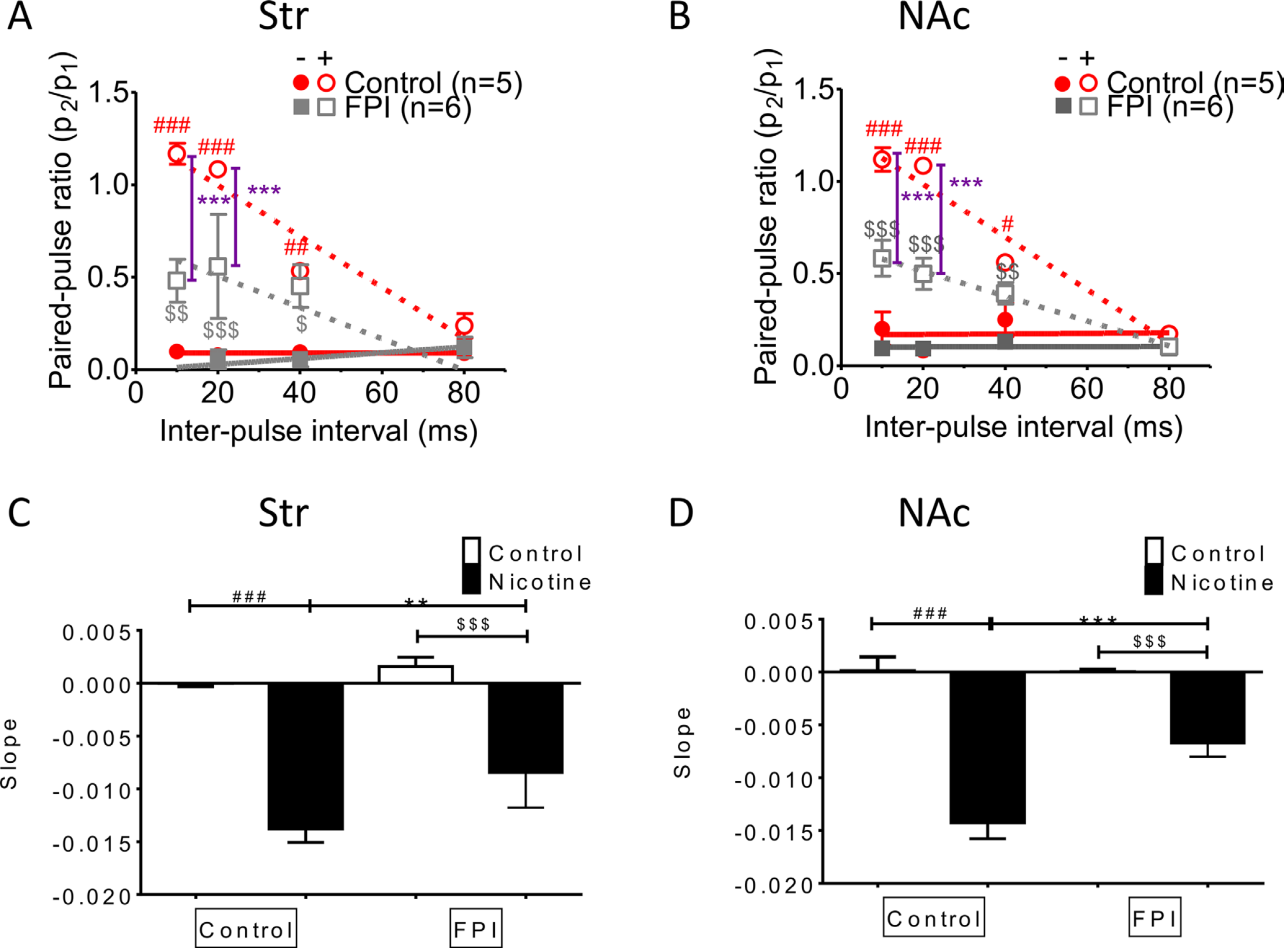
Nicotine desensitization–related paired-pulse facilitation at a high frequency was suppressed after FPI The facilitation was suppressed in both the striatal (**A**) and NAc shell portion (**B**). (**C**) The slope revealed that nicotine desensitization related to facilitation declined with stimulation interval prolongation in the striatum, and this short-term facilitation was diminished after FPI. (**D**) A similar result was found in the NAc shell portion. (DA concentration: one-way ANOVA followed by a Bonferroni post hoc test for multiple comparisons, Control + Nicotine vs. FPI + Nicotine, *P* < 0.001^***^; Control vs. Control + Nicotine, *p* < 0.05^#^, *p* < 0.01^##^, *p* < 0.001^###^; FPI vs. FPI + Nicotine, *p* < 0.05^$^, *p* < 0.01^$$^, *p* < 0.001^$$$^).

Paired-pulse facilitation declined with frequency in both striatum and NAc shell slices infused with nicotine, as documented previously [30]. This phenomenon was suppressed by FPI (Figure 5A and 5B; control with nicotine infusion: red open circle, FPI with nicotine infusion: gray open square). The slope revealed that facilitation related to nicotine desensitization declined with stimulation interval prolongation in the striatum and that this short-term facilitation was diminished after FPI (Figure 5C); a similar result was observed in the NAc shell portion (Figure 5D).

## DISCUSSION

Habit formation is thought to be related to striatal activity [8, 31], whereas activation of the mesolimbic DA system is central to associative learning, reinforcement, and drug addiction [30, 32, 33]. There is evidence that drug addiction involves multiple functional areas, including the mesolimbic dopaminergic pathway [33], which processes reward-related stimuli; the amygdala [34], which generates emotions; the hippocampus, which facilitates learning and memory creation [35]; and the prefrontal cortex, which underlies executive action [36]. Furthermore, all these process are governed by the corticolimbic network [37].

In chronic smokers, the upregulation of nAChR subtypes may be induced after long-term exposure to nicotine [38, 39], which indicates that the number of high-affinity nicotine binding sites in multiple regions of the brain has increased [40–42]. Furthermore, higher nicotine exposure may cause rapid nAChR desensitization, which induces receptor function loss. Subsequently, after long-term desensitization, upregulation may be promoted to compensate for the weakening signal from the inactivated receptors [43, 44]. These changes may induce higher sensitivity to nicotine and are related to nicotine addiction [45]. A previous study indicated that DA transmission was affected after a head injury, and our previous data indicate that FPI suppressed DA release in both the striatum and NAc region [46–49].

A nationwide population-based cohort study revealed that TBI increases the risk of developing a substance-related disorder (SRD) [50, 51]. Addiction has been linked to dysfunctions in the DA system [52, 53], and chronic drug exposure was reported to suppress tonic DA levels, increase phasic DA release, and promote peculiar stimulus–reinforcer associations, all leading to the development of addiction [54]. The nature of the relationship between TBI and a substance-related disorder or addiction, particularly smoking, is controversial, and the role of nicotine desensitization in this behavioral process has yet to be determined.

In this study, we determined that DA release was suppressed in the FPI rat brain slices, and that the proportion of suppression related to nicotine desensitization remained after FPI. The I/O curve shifted to the right, with a weaker response signal evoked in the DA system after FPI, but nicotine desensitization and a reinforcing signal remained. This indicates that brain injury suppressed DA release, and a response to reward required a significantly stronger stimulus. Moreover, nicotine-related DA release patterns were altered in different ways. We determined that striatal DA release was suppressed by FPI, but significant frequency augmentation and concentration differences existed between tonic and phasic release related to nicotine infusion. By contrast, DA release in the NAc shell portion was reduced after FPI, and further suppression related to nicotine desensitization was observed. The differences between tonic and phasic release related to nicotine infusion were predominant in the NAc shell but were diminished after FPI.

Although the role of phasic DA release in drug abuse has been emphasized [54, 55], we believe that the difference between tonic and phasic release may be critical in increasing the “signal-to-noise ratio” of DA signaling. Enhancements in DA signal contrast have been interpreted as an enhancement of motivation in drug cravings [56] and potentiation of drug-related stimuli [57]. Moreover, they provide a “prediction-error teaching” signal that reinforces addictive behaviors [58]. Thus, a difference was observed between tonic and phasic DA release (Figure 4), which may be another means through which the signal-to-noise ratio is enhanced in order to facilitate addiction. DA release related to nicotine desensitization and the differences between tonic and phasic release remained after PFI (Figures 1 and 4).

Although DA release was suppressed after head injury, reward behavior is difficult to initiate, and a higher dosage of the substance has been necessary to maintain the behavior [59, 60]. Subsequently, once the reward behaviors are initiated and maintained using the higher dosage, significant withdrawal and abstinence symptoms may result. Cravings and aberrant behaviors related to cue–stimuli associations may be initiated and promoted because of the enhanced signal-to-noise ratio in DA release after a chronic drug experience [54] Augmenting phasic DA release after a drug experience not only reinforces the stimulus associations already made [59, 61] but is also associated with learning procedures [60, 62, 63]. Therefore, the phenomena could be explained of why patients who are previous smokers and substance abusers are more likely to continue smoking and substance abuse.

In summary, because of the suppression of DA release by FPI and nicotine desensitization, DA release was observed to be lowered by FPI in this study, and stronger stimulation was required to induce phasic DA release. The enhanced deviation between tonic and phasic DA release augments the signal-to-noise phenomenon that might enhance reinforcing information under the exposure of stimuli, which in turn may explain why patients with head injury may require more powerful stimuli. The induction of higher phasic DA release and the difference between tonic and phasic DA release may be the reason why an epidemiologic report indicated that TBI increases the risk of SRDs [50].

## MATERIALS AND METHODS

### Animals and fluid percussion injury

Sprague Dawley rats (6 weeks old) weighting 200–250 g were randomly subjected to either sham injury (*n* = 36) or fluid percussion injury (6 ± 0.2 psi, *n* = 36). Animals were provided food and water ad libitum and were housed in a 12 h light-dark cycle room. The fluid percussion injury was performed while rats were anesthetized with Tiletamine-Zolazepam (50 mg/kg, i.p.; Zoletil, Vibac, France) and xylazine (10 mg/kg, Rompun, Bayer, Germany). A fluid percussion device (model HPD-1700, Dragonfly R&D) was used to produce TBI. The injury was induced by striking a piston with a weighted metal pendulum released from a pre-determined angle (mild TBI 16°, more severe TBI 43°). Using a pressure transducer coupled to a digital real-time oscilloscope (TDS210, Sony Tektronix Corp., Osaka, Japan), the pressure pulses were recorded and measured extra-cranially, and then analyzed by WaveStar software (Sony Tektronix Corp.) in order to convert injury intensity to pounds per square inch (psi) of overpressure based on prior instrument calibration. The fluid percussion device delivered transient pressure fluid pulses with constant waveform and duration (17–21 ms). Sham- injured animals underwent surgical preparation and connection to the machine without administration of FPI.

### NAc and striatal brain slice preparation

Brain slices were prepared as described previously [64, 65], after the rats were decapitated. Specifically, the brains were placed into a beaker filled with oxygenated (95% O_2_/5% CO_2_) cold cutting solution containing 194 mM sucrose, 30 mM NaCl, 4.5 mM KCl, 1 mM MgCl_2_, 1.2 mM NaH_2_PO_4_, 10 mM glucose, and 26 mM NaHCO_3_. The tissue blocks containing the NAc and striatum were cut into coronal section slices (280 μm) within a chamber filled with cold cutting solution by using a tissue slicer (VT 100, Leica). These tissue slices were then transferred to a holding chamber filled with oxygenated artificial CSF solution (aCSF; 126 mM NaCl, 3 mM KCl, 1.5 mM MgCl_2_, 2.4 mM CaCl_2_, 1.2 mM NaH_2_PO_4_, 11 mM glucose, and 26 mM NaHCO_3_) at 31°C for 20–30 min.

### FSCV recording for dopamine release assessment and nicotine infusion

FSCV recording was performed as described previously [28, 46]. After slices were transferred into the chamber (at 31–33°C) filled with aCSF at a 2 mL·min perfusion rate, a custom-made carbon fiber (7 μm in diameter; Goodfellow Corp., Oakdale, PA, USA) was lowered to a depth of 100 μm into the NAc under stereoscopy. To stabilize the background current, the potential of the carbon fiber was increased from −0.4 V to 1.0 V and then reduced to −0.4 V using a triangular waveform (400 V/s, 7 ms in duration) applied every 100 ms. A 5-s (50-scan) control period was applied to the carbon fiber. The DA peak oxidation currents were then digitally subtracted from those obtained during the peak of the response following electrical stimulation, and all signals used in the statistical analyses matched the expected voltammetric profile for DA [66]. The current signals were then converted to DA concentrations using a calibration performed for each electrode using a 1-μM DA standard solution. To assess the capacity of axon terminals for releasing DA during stimulation, two types of voltammetric signals were obtained at each recording site by using a single pulse (for tonic) and 10 pulses (for phasic) delivered at 25 Hz under different stimulation intensities (from 1 V to 10 V). After tonic and phasic DA signals were obtained with different stimulation intensities at each site, the values were summed, averaged, and plotted in an input/output (I/O) curve. For the nicotine desensitization experiments, the nicotine tartrate (N5260, Sigma Aldrich, St. Louis, MO) 1μM was infused for twenty minutes after the dopamine signal were stabilized.

### Statistical analyses

Data in the text and figures are expressed as means + standard errors of the mean (SEMs). Statistical analyses of data for curves of DA release I/O, stimulated bursting frequencies, multiple HFS trains, and the paired-pulse ratio were performed using two-way analysis of variance (ANOVA) followed by a Bonferroni post hoc test for multiple comparisons. One-way ANOVA and a Bonferroni post hoc test were used to determine DA release through the change in nicotine and the slope bar chart. All statistical tests were two tailed and were performed using GraphPad Prism 5.02 (GraphPad Scientific, San Diego, CA, USA); *p* < 0.05 was considered significant for all analyses. The statistical details for all Figures 1–5 are included below after the Figure Legends.

## CONCLUSIONS

DA release in the striatum and NAc shell is significantly suppressed after FPI, and nicotine desensitization–related DA tonic release suppression, frequency-dependent augmentation, and HFS-related gating release in both the striatum and NAc shell are present but attenuated after FPI. Furthermore, nicotine desensitization–induced differences between phasic and tonic release for reward-related signals become less prominent in the shell portion after FPI. Therefore, FPI adversely affects DA release from the NAc and dorsal striatum, although reward-related signals can still be produced if higher DA concentration differences are induced using more intense stimuli.

## Abbreviations

aCSF: artificial cerebrospinal fluid
ANOVA: analysis of variance
Ctx: Cortex
DA: dopamine
FPI: fluid percussion injury
FSCV: fast-scan cyclic voltammetry
HFS: high-frequency stimulation
I/O curve: input/output curve
Mec: mecamylamine
NAc: nucleus accumbens
nAChR: nicotine receptor
Psi: pound per square inch
SEMs: standard errors of the mean
SRDs: Substance-related disorders
Str: Striatum
TBI: traumatic brain injury
